# Soluble Sugar and Lipid Readjustments in the *Yarrowia lipolytica* Yeast at Various Temperatures and pH

**DOI:** 10.3390/metabo9120307

**Published:** 2019-12-17

**Authors:** Varvara Yu Sekova, Daria I. Dergacheva, Elena P. Isakova, Natalya N. Gessler, Vera M. Tereshina, Yulia I. Deryabina

**Affiliations:** 1 A.N. Bach Institute of Biochemistry, Russian Academy of Sciences, bld 33-2, Leninsly Prospect, Moscow 119071, Russia; ddarya1993@gmail.com (D.I.D.); gessler51@mail.ru (N.N.G.); yul_der@mail.ru (Y.I.D.); 2 Winogradsky Institute of Microbiology, Research Center of Biotechnology of the Russian Academy of Sciences, Leninsky Ave. 33/2, Moscow 119071, Russia; v.m.tereshina@inbox.ru

**Keywords:** thermal shock, ambient pH, yeast, metabolic readjustments, lipids, carbohydrates

## Abstract

Microorganisms cope with a wide range of environmental challenges using different mechanisms. Their ability to prosper at extreme ambient pH and high temperatures has been well reported, but the adaptation mechanism often remains unrevealed. In this study, we addressed the dynamics of lipid and sugar profiles upon different cultivation conditions. The results showed that the cells grown at various pH and optimal temperature contained mannitol as the major cytosol sugar alcohol. The elevated temperature of 38 °C led to a two- to three-fold increase in total cytosol sugars with concurrent substitution of mannitol for trehalose. Lipid composition in the cells at optimal temperature changed insignificantly at any pH tested. The increase in the temperature caused some drop in the storage and membrane lipid levels, remarkable changes in their composition, and the degree of unsaturated fatty acids. It was shown that the fatty acid composition of some membrane phospholipids varied considerably at changing pH and temperature values. The data showed a pivotal role and flexibility of the sugar and lipid composition of *Y. lipolytica* W29 in adaptation to unfavorable environmental conditions.

## 1. Introduction

The *Yarrowia lipolytica* yeast is capable of adapting to various environmental challenges. The microorganism can prosper at extremely high (up to 9.5) and low (up to 2.5) [1] ambient pH [2], as well as under conditions of high salinity using either dry or hydrophobic substrates [3]. Alkaline tolerance is not common among the yeast species since their optimal growth pH is usually within the range 5.0–6.5, and the yeast can rarely resist alkaline stress of above pH 8.0. The *Y. lipolytica* yeast has been widely used for producing lipase, organic acids, and some recombinant proteins [4,5]. The ability of *Y. lipolytica* to utilize low-cost substrates of various compositions (petroleum waxes, crude biomass hydrolysates, and industrial waste) and to yield a large amount of biomass renders the yeast species a most prospective one for biotechnological use.

The ability of living organisms to survive under stress is usually associated with changes in gene expression, resulting in some readjustments at the molecular and biochemical levels. External effects, such as temperature, ambient pH, and salinity, greatly influence the growth and development of a yeast cell. The adaptation response to changes in medium temperature and salinity besides adaptive synthesis of stress proteins includes the altered content of membrane lipids, accumulation of some cytosol carbohydrates, and cytoprotectant molecules [6]. The osmolytic system is known to be an essential defense factor against various harmful effects. The carbohydrate composition in fungal cells changes depending on the culture growth phase and physiological state. Thus, environmental challenges, such as thermal or osmotic shocks, and oxidative stress can promote either accumulation or consumption of certain carbohydrates, namely non-reducing disaccharide trehalose and two polyols, glycerol and *D*-arabitol, in a cell [7].

There is a group of carbohydrate compounds, such as disaccharide trehalose, glycosyl-glycerol, and some polyols (glycerol, erythritol, arabitol, mannitol, inositol, sorbitol), which protect microorganisms against various stresses. These metabolites can stabilize macromolecules and cell membranes under the conditions of water shortage, osmotic shock, and both high and low temperatures [8,9]. A number of the compounds in the cells can perform osmo-protective and thermo-stabilizing functions [10]. In *Candida albicans*, an osmotic shock of 1 M sodium chloride induced a minor decrease in trehalose and *D*-arabitol contents. However, oxidative exposure by the oxidants promoted a significant rise in both trehalose and *D*-arabitol levels [10]. Under cold and osmotic stresses, the *Aspergillus niger* fungus showed the excretion of glycerol, *D*-arabitol, and erythritol into the medium [11]. A high erythritol level was also observed in *Y. lipolityca* at extremely low pH (pH 3.0) [12]. Some soluble cytosol carbohydrates function as direct antioxidants on cellular structures and macromolecules. Thus, mannitol, widely known as an reactive oxygen species (ROS) scavenger, is capable of protecting animal and plant pathogens against ROS generated by the host organism during the anti-stress response [13,14]. The participation of mannitol in the antioxidant defense response of a fungal cell has also been reported for plant pathogens of *Alternaria alternata* [15] and *Uromyces fabae* [16].

Natural disaccharide trehalose, which acts as a universal signaling and protective agent in a fungal cell as a protein and phospholipid stabilizer in the membrane lipid bilayer, also appeared to be a powerful antioxidant. So, *S. cerevisiae* demonstrated that increases in trehalose levels led to a higher survival during mild heat shock (38 °C), after the ROS-generating system (2 mM H_2_O_2_ and 1 mM FeCl_3_ at 28 °C) was exposed, and administration of the proteasome inhibitor (MG132) to the medium [17]. Similar results were published in the paper [18] concerning the study of metabolic rearrangement in *S. cerevisiae* under prolonged heat exposure (8 h at 37 °C). The authors observed a jump in the trehalose content after 0.5 and 2 h of thermal exposure, with further stabilization of the disaccharide level. Moreover, the *tps1* and *tps2* mutants of *S. cerevisiae*, unable to synthesize trehalose, were much more sensitive to ROS effects than the wild type, which indicates the high antioxidant role of trehalose in a yeast cell [19]. Cell membranes, as a defense barrier of a cell, alter their lipid composition in response to various kinds of stress. Alkaliphilic micromycete of *Sodiomyces tronii* contain more sterols and sphingolipids in the membrane lipids at acidic ambient pH [20]. Non-optimum pH invoked significant changes in the degree of fatty acid unsaturation, amount of the sterols, and phosphatidylcholine (PC) fractions in the membrane lipids in alkaliphilic *Sodiomyces magadii* and *alkalinus*, as well as alkali-tolerant *Acrostalagmus luteoalbus* and *Chordomyces antarcticus* [20].

Moreover, the intrinsic flexibility of the eukaryotic lipidome depending on growth conditions, such as temperature and growth phase, was shown using *Saccharomyces cerevisiae* [21]. Ethanol stress in the *S. cerevisiae* yeast led to some changes in membrane structure and lipid composition [22]. Some other factors, namely, carbon source, can impact considerably on the lipid composition and degree of unsaturation of the fatty acids [23]. Some recent studies have assayed the influence of sterol composition on the survival of *S. cerevisiae* at various stresses. Hence, the survival of the mutant in *erg6Δ*, unable to synthesize ergosterol, remarkably dropped after osmotic shock [24]. Some osmolytes, in particular, trehalose, play an essential role in protecting fatty acids against free radical oxidation. They can prevent the dehydration of unsaturated linoleic and linolenic acids, resulting in aldehyde formation and inhibition of the auto-oxidation of unsaturated fatty acids, leading to peroxide formation. It all together indicates a direct interaction of trehalose with acyl chains and stabilization of fatty acid structures [8]. Inositol also plays a significant role in cell protection, being a precursor of phosphatidylinositol (PI), which, in turn, is the source for such necessary signaling and structural molecules as phosphoinositides, inositol polyphosphate [25], and inositol-containing sphingolipids (SL) [26]. Moreover, some genes of glycerophospholipid synthesis maximally express when no inositol is in the growth medium, and repress when inositol is added to that [27].

The content and composition of lipids and fatty acids in the *Y. lipolytica* cells significantly depends on the growth conditions and the type of substrate used. In studies of the lipid and fatty acid composition using *Y. lipolytica Po1 g* grown on various substrates (in defatted rice hydrolyzate, the cells could accumulate up to 81.5% of neutral free fatty acids, 6.12% of monoacylglycerides (MAGs), 5.32% of triglycerides (TAGs), and only 4.87% of diacylglycerides (DAGs). The dominating free fatty acid were oleic (C18:0, 55.55%), palmitic (C16:0, 17.76%), palmitoleic (C16: 1, 14.62%), and stearic (C18:0, 4.39%) acids. However, long-chain fatty acids (<C18:0) made up only 8.27% of the fatty acids [28].

Genetic manipulations help to change the lipid composition in *Y. lipolytica* cells. Thus, the commercial production of ω-3 oils using *Y. lipolytica* yeast has high industrial capacity, which has prompted researchers to modify the synthesis of some long-chain polyunsaturated fatty acids using genetic engineering. Some long-chain fatty acids, namely eicosapentaenoic (EPA, C20:5 n-3) and docosahexaenoic acids (DHAs, C22:6 n-3), are used for medical purposes for patients with diseases of the cardiovascular system and hypertriglyceridemia [28]. This acid plays an important role in the treatment of some diseases, like cancer prevention, antiatherogenic effects, and so on. The *Y. lipolytica* yeast grown on soybean as a substrate reached a conjugated linoleic acid (CLA) yield of 3.1 g/L (16% dry weight), as well as 0.9 g/L of release into the culture medium [28].

Changes in the cultivation conditions cause a complex readjustment of the fatty acid composition in yeast cell and the degree of unsaturation. Thus, the application of Se^4+^ (20 mg/L) into the culture medium increased the unsaturated fatty acid level, especially oleic (C18:1), linoleic (C18:2), linolenic (C18:3), and palmitoleic (C16:1) acids in *Candida utilis* [29]. Moreover, the authors showed an increase in the margaric (C17:0), heptadecenoic (C17:1), and tetradecanoic (C14:1) acid levels. In these conditions, the increase in the unsaturated fatty acids share in the lipid profile in the yeast cell was related to an increase in desaturase activity, which increased the cell membranes fluidity in the yeast [29]. Some changes in the growth conditions and especially temperature shifts could determine the fatty acids composition in yeast cell, because the fatty acids alter the membrane fluidity [30]. In [31], a three-fold increase in α-linolenic acid production under low-temperature cultivation in the *Y. lipolytica* line with transformed Δ12–15 desaturase (RkD12–15) was shown. So far, some studies have used low temperature cultivation in some yeast species, namely *Y. lipolytica* and *Mortierella alpina*, to produce polyunsaturated fatty acids [32,33]. When cultivated at 28 °C for 24 h followed by 20 °C, *Y. lipolytica* could increase γ-linolenic acid production by 60.9% [32]. The elevating temperature increased the long-chain fatty acid levels in *Y. lipolytica* yeast. This led to the disappearance of short-chain fatty acids like capric acid (C10:0) [28]. Therefore, the cultivation conditions can help to control fatty acids synthesis in yeast cells.

The presented changes in the carbohydrate and lipid profiles in a fungal cell under various stresses and their mutual interaction suggest that these compounds should play a vital role in the adaptation mechanism of the yeast. In this regard, in the present study, we show the adaptive metabolic remodeling of the *Y. lipolytica* yeast under different pH and temperatures values.

## 2. Results

### 2.1. Growth of the Y. lipolytica Yeast under Different Conditions

**Figure 1:**
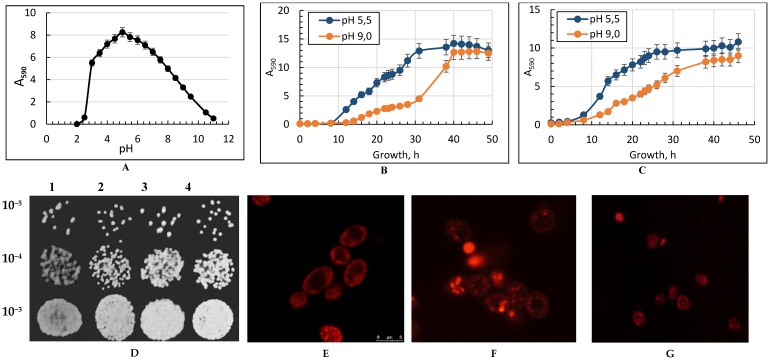
The effect of ambient pH (panel **A**) on the growth of *Yarrowia lipolytica* W29 in glycerol-containing (1%) medium. Panels **B**,**C**—Growth curves of the *Y. lipolytica* yeast grown at 29 °C (panel **B**) and at 38 °C (panel **C**) in glycerol-containing medium. Absorbance was assessed every two hours in cell suspension at the wavelength of 590 nm (A_590_) using a spectrophotometer. Error bars represent the standard deviation of triplicates. Mean values are displayed (*n* = 3, ±SD). Panel (**D**)—Spot dilution assays in yeast peptone dextrose (YPD) medium (10 μL per spot). Yeast cells were spotting on corresponding positions of different plates by serial dilutions: **1**—pH 5.5; 29 °C; **2**—pH 9.0; 29 °C; **3**—pH 5.5; 38 °C; **4**—pH 9.0; 38 °C. The plates were incubated at 29 °C for 48 h. Panels (**E**–**G**)—micro images of the potential-dependent stain of the *Y. lipolytica* cells with rhodamine 123 (Rh123). (**E**)—pH 5.5; 29 °C; (**F**)—pH 5.5; 38 °C; (**G**)—pH 9.0; 38 °C. The cells were incubated with 0.5 µM Rh123 and examined after 30 min. Incubation medium contained 0.01 M phosphate buffer saline (PBS) and 1% glycerol, pH 7.4. The regions of high mitochondrial polarization are bright red due to the concentrated dye. To examine the Rh123-stained preparations, filters, 02, 15 (Zeiss), were used (magnification ×100). The photos were taken using an AxioCam MRC camera.

The yeast was grown at various ambient pH values. Results demonstrate the maximal linear growth at pH 5.5 and no growth at pH below 3.0 (Figure 1A) and above 10.5 (data not shown). Figure 1A shows the dependence of the cultivation pH at the optimal temperature on the growth rate of *Y. lipolytica*. The optimum pH of the yeast growth proved to be within 4.5 and 6.0. The growth at ambient pH ranged from 3.5 to 4.5 and showed a smooth rise on the curve, followed by a slight jump at pH 5.0 and a gradual decrease in the growth rate at pH 10.5 to 11.0. We chose two values for further experiments: 5.5 and 9.0. Besides, we cultivated the *Y. lipolytica* yeast at different temperatures from 20 to 40 °C with a step of two degrees. At the optimal pH of 5.5, the yeast grows well in a wide range of temperatures from 20 to 40 °C, showing maximal growth at 29 °C (Figure 1B). *Y. lipolytica* does not grow at a temperature above 40 °C. At temperatures of 38 to 40 °C, the growth rate significantly decreased, and the temperature above 40 °C blocked the growth (data not shown). Increasing the pH to 9.0 in the optimal temperature resulted in a more extended log-phase to 40 h and some decrease in growth. The effect of the combined influence led to a significant (about 40%) decline in the yield biomass compared to that under the optimal conditions (Figure 1C).

To evaluate the physiological state of the *Y. lipolytica* cells under stress, we assayed the key antioxidant enzymes, namely superoxide dismutases (SODs) and catalases (CATs), in different growth conditions, including the stress ones. The results are presented in Table 1. Increasing the pH led to a 5.8-fold rise in SODs activities, whereas the elevated temperature increased 12.2-fold. Under the combined stress, those activities increased by 10 times (Table 1). The CATs activity changed insignificantly at increasing ambient pH and dramatically rose by some orders at both thermal and combined stress (Table 1).

The assay of the reduced and oxidized glutathione level showed that increasing the pH halved the GSH content while the GSSG level remained unchanged (Table 1). However, the elevated temperature increased the GSH level three-fold and the GSSG level 10-fold. The redox potential of HSSG/GSH increased up to 0.48 at alkaline pH and 0.67 at the elevated temperature compared to 0.24 under the optimal conditions (Table 1). However, the ratio of the reduced glutathione to the oxidized one decreased more than two-fold. The obtained data indicate the development of the anti-stress response by the *Y. lipolytica* yeast under these conditions. Thus, we established a few parameters for the growth of the yeast: pH 5.5, 29 °C. The ability of the *Y. lipolytica* to grow at different pH and above-optimal temperatures renders the species a convenient model for studying lipid and sugar contents in response to various pH and temperature values.

### 2.2. Cell Viability and Vitality Assays

The survival assessment by the spotting test as well as the quantitative assay of live, dead, and budding *Y. lipolytica* cells grown under various conditions showed that the viability of yeast cells changed insignificantly in all the versions and remained at a rather high level within 75% and 95% (Figure 1D). The number of budding cells made up, on average, 20% of the total number. Potentiometric staining of cells with Rhod 123 demonstrated a high degree of yeast mitochondria energization under both optimal (Figure 1E) and stress conditions, namely the elevated temperature (Figure 1F) and combined stress (Figure 1G). It should be noted that the cell shape and size depend on the cultivation conditions. When grown at the elevated temperature and combined stress, the cell decreased in size by 15% and 50%, respectively.

These results indicate a high energy status of yeast cells under all the conditions tested.

### 2.3. Cytosolic Soluble Carbohydrate Analysis

**Figure 2:**
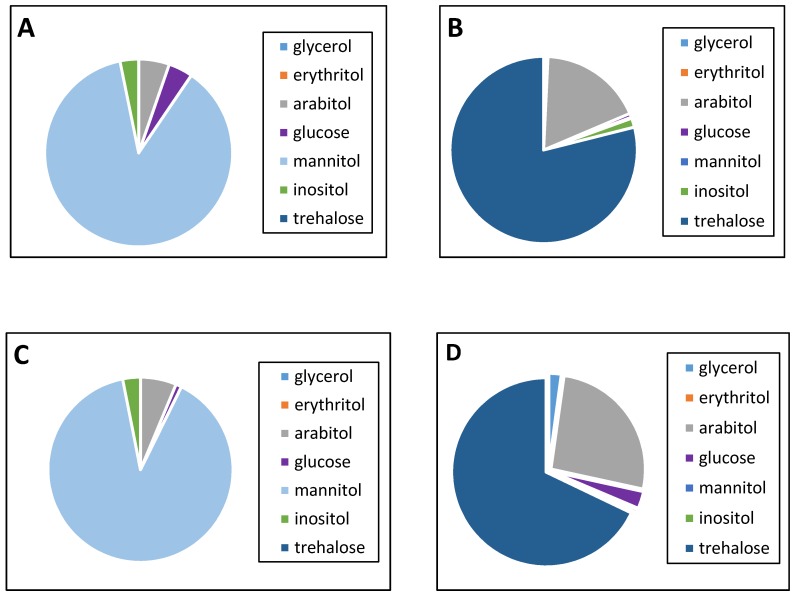

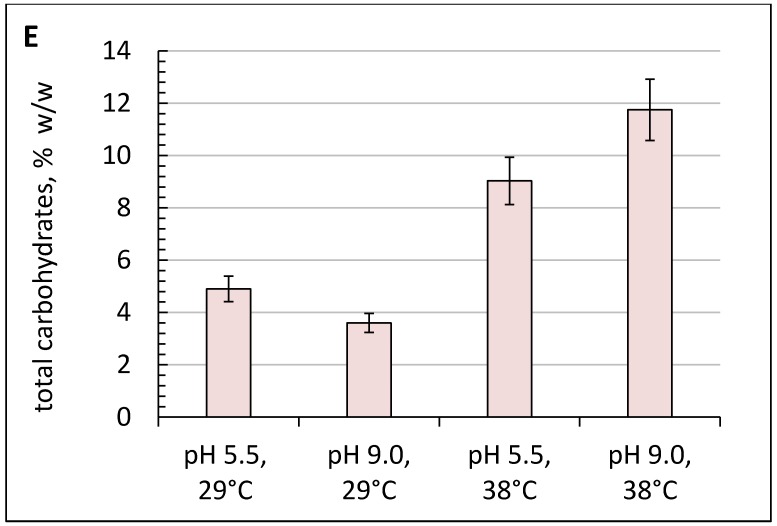
The cytosol sugar and polyol composition in the *Y. lipolytica* yeast grown under different conditions. The share of each soluble cytosolic sugar fraction: (**A**)—pH 5.5, 29 °C; (**B**)—pH 5.5, 38 °C; (**C**)—pH 9.0, 29 °C; (**D**)—pH 9.0, 38 °C. (**E**)—total carbohydrates and polyols content. Error bars represent the standard deviation of triplicates. Mean values are displayed (*n* = 3, ±SD). a—*p* < 0.05; b—*p* < 0.03.

The changes in the cytosolic sugar composition and content can serve as the defense response to stress. So, next, we tracked the composition of sugars and lipids under various culture conditions. Thus, under optimal growth conditions (pH 5.5; 29 °C), the sugar content made up nearly 5% of the dry weight in the *Y. lipolytica* cytosol (Figure 2E). A pH increase to 9.0 (Figure 2E) declined the sugar amount by 25%. At the optimal temperature, mannitol was dominating and reached 87% to 89% of the total cytosol carbohydrates at any pH tested (Figure 2A,C). *D*-arabitol, inositol, and glucose, as the minority, exceeded no more than 6% of the total amount. Moreover, any changes in ambient pH caused no sharp fluctuations in the cytosolic sugar composition. A pH increase to 9.0 led to a rise in the arabitol fraction (by 20%) while the glucose level decreased nearly four-fold (Figure 2C). Neither the growth substrate of glycerol used nor the “stress” sugar of trehalose was revealed in the repertoire of the identified carbohydrates in the yeast cytosol.

An increase in cultivation temperature caused the most remarkable changes in cytosol sugars. Unlike the optimal conditions (pH 5.5; 29 °C), the elevated temperature and especially, the combined stress (alkaline pH (pH 9.0) and elevated temperature (38 °C) caused a significant more than twice, increase in the total sugar content (Figure 2D,E), including a 10-fold increase in the arabitol fraction. Some glycerol amount (of about 5% of the total sugar content) was observed (Figure 2D). In the cells grown at 38 °C, trehalose reached 65% to 70%, and *D*-arabitol 20% to 25%, dominating among the carbohydrates, while mannitol disappeared and the minor carbohydrates (Figure 2B,D) went down even more.

### 2.4. Membrane and Storage Lipids Profile at Various Ambient pH and Temperatures

**Figure 3:**
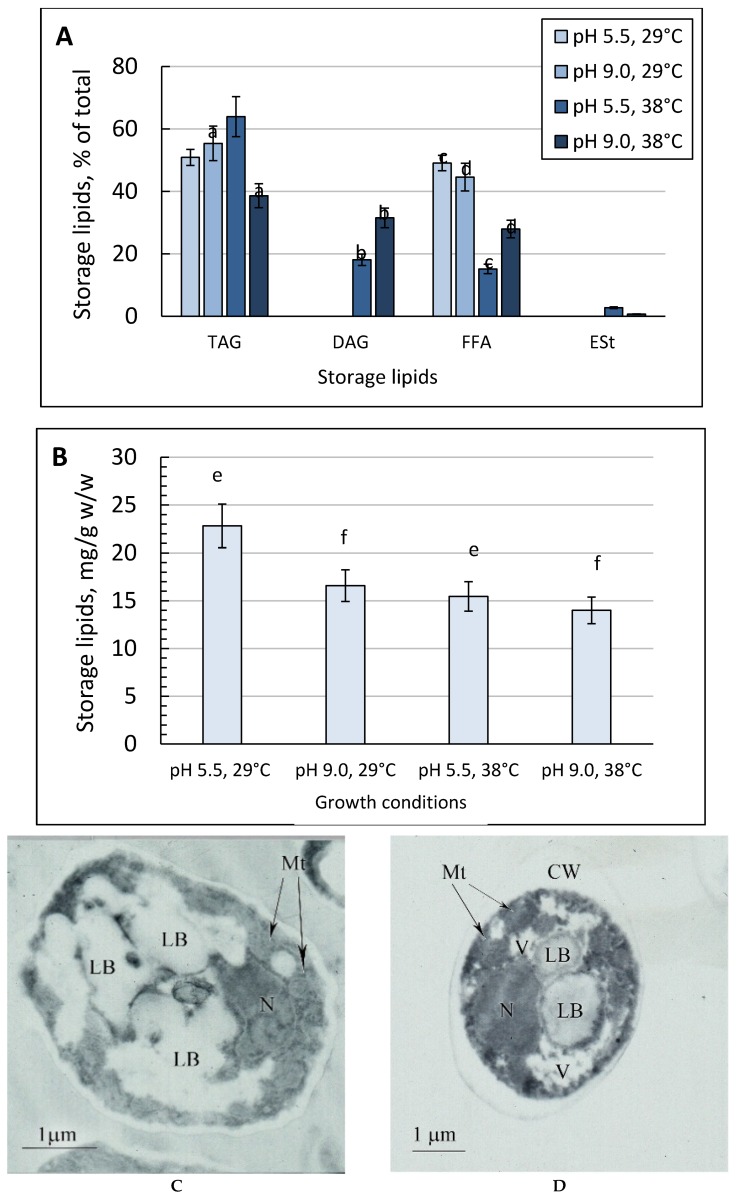

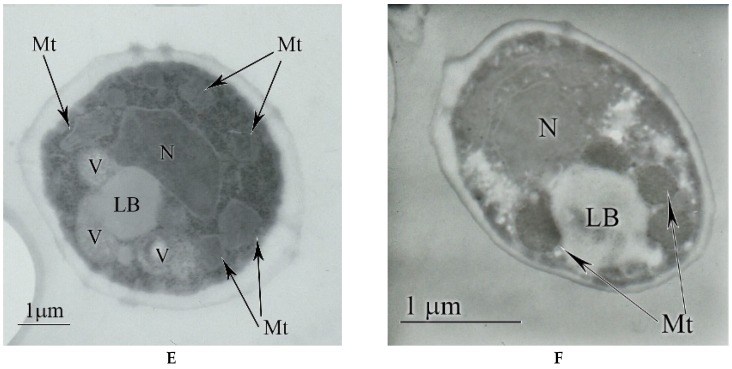
The storage lipid composition in the *Y. lipolytica* yeast grown under different conditions. (**A**)—the share of each storage lipid fraction, %; (**B**)—the total storage lipids content, mg/g *w*/*w*. TAG—Triacylglycerols; DAG—Diacylglycerols; FFA—Free fatty acids; ESt—sterol esters. Error bars represent the standard deviation of triplicates. Mean values are displayed (*n* = 3, ±SD). (**C**–**F**)—Transmission electron microscopy of *Y. lipolytica* W29 cells grown under various conditions: (**C**)—pH 5.5, 29 °C; (**D**)—pH 9.0, 29 °C; (**E**)—pH 5.5, 38 °C; (**F**)—pH 9.0, 38 °C; CW—cell wall; LB—lipid body; Mt—mitochondria; N—nucleus; V—vacuole. a—*p* < 0.04; b—*p* < 0.01; с—*p* < 0.001; d, e—*p* < 0.02; f—*p* < 0.002.

In some recent studies, the influence of growth conditions, including carbon source on storage lipid composition, was shown [34]. At the optimal growth temperature, storage lipids were mainly comprised of TAG and FFA in equal amounts (Figure 3A), and the changes in ambient pH did not affect their ratio. The storage lipid level decreased by a third at pH 9.0 (by 27%) (Figure 3B). The increase in the growth temperature to 38 °C declined the total storage lipids content by 35% (Figure 3B), and the FFA fraction decreased by 4.8 and 1.9 times at pH 5.5 and 9.0, respectively (Figure 3A). In the lipid profiles at pH 5.5, DAG (18%) appeared, which reached 31.5% of the total storage lipids at pH 9.0 (Figure 3A). At pH 5.5, the TAG level reached 63% (Figure 3A) while a pH increase to 9.0 declined to 37% of the total storage lipid (Figure 3A). At any pH, the thermal shock led to the appearance of some sterol esters (ESt) in the storage lipids (Figure 3A). The results were confirmed by the ultra-structural features of the yeast cells grown under stress conditions (Figure 3C–F). The largest amount of the storage lipids as lipid bodies (LB) was revealed in the optimal conditions (Figure 3C), decreasing under the stress ones (Figure 3D–F). Under any kind of stress, in the cells, there were some lipid bodies associated with the nucleus. Under the thermal shock, the structure was associated with mitochondria located around lipid bodies, forming the complex of “lipid bodies + nucleus + mitochondria”. It could indicate an active migration of lipids between the organelles, maintaining the energy cell state under stress (Figure 3D–F).

**Figure 4:**
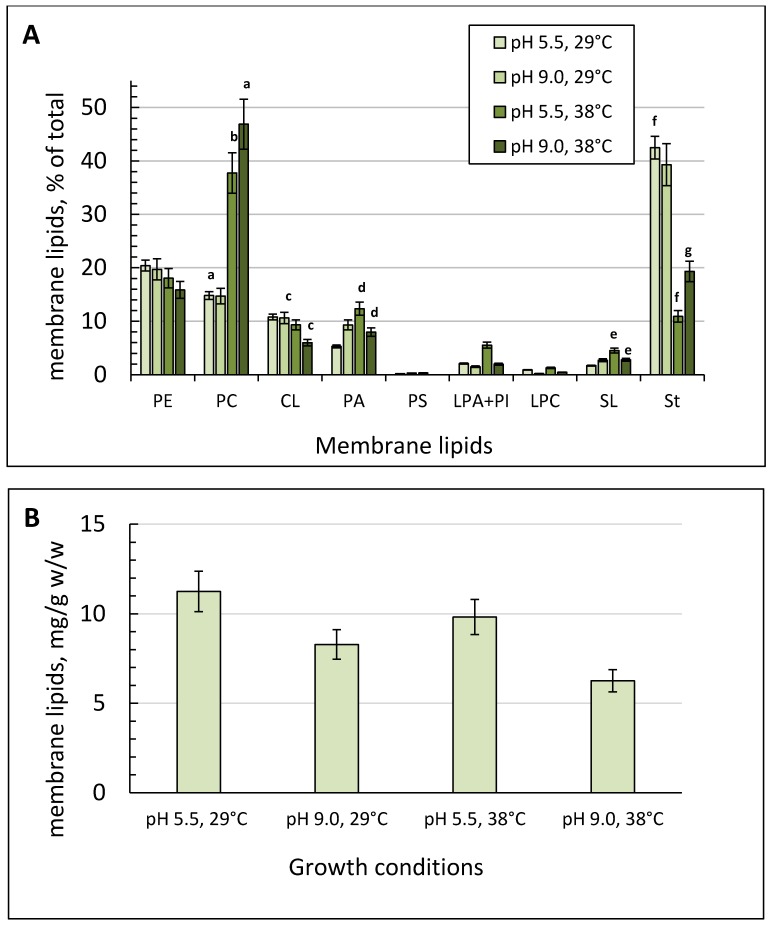
Membrane lipid composition of *Y. lipolytica* under different conditions. (**A**)—the share of each membrane lipid fraction, %; (**B**)—the total membrane lipid content mg/g. PE—Phosphatidylethanolamines; PC—Phosphatidylcholines; CL—Cardiolipins; PA—Phosphatidic acids; LPA + PI—lysophosphatidylethanolamine + Phosphatidylinositols; LPC—Lysophosphatidylcholines; SL—Sphingolipids; St—Sterols. The conditions of the culture growth are indicated in the panels. Error bars represent the standard deviation of triplicates. Mean values are displayed (*n* = 3, ±SD). a—*p* < 0.01; b, e, f—*p* < 0.02; c—*p* < 0.03; d, g—*p* < 0.05.

The membrane lipid amount reached its peak of 12.98 mg/g dry weight under the optimal conditions (Figure 4B). Elevating pH at the optimal growth temperature led to some decrease in the membrane lipid content by 36% (Figure 4B). The membrane lipid composition of the culture grown at 29 °C was the same at both pH values tested. In the membranes, phospholipids (about 50%) and sterols (St, 45%) dominated, with a minority of SL. The main phospholipids were phosphatidylethanolamine (PE, 16–20%), phosphotidylcholine (PC, 15%), and cardiolipin (CL, 7–9%) (Figure 4A). Phosphatidic acids (PA) varied from 5% at pH 5.5 to 9% at pH 9.0 (Figure 4A). The minority comprised lysophosphatidylethanolamines (LPE), lysophosphatidylcholines (LPC), and phosphatidylinosites (PI).

A temperature increase to 38 °C resulted in nearly a 30% decrease in the total membrane lipids (Figure 4B) partly due to the more than three-fold decrease in the St level (from 45% to 11%). However, the PE fraction remained at the same level while the PC and PA fractions were 2.0 to 2.5 times increased (Figure 4A). The minority remained constant except for the SL fraction, which was 2.6 times more at the elevated temperature and optimal pH (Figure 4A).

The combined stress caused the most significant changes in both the amount and composition of membrane lipids, resulting in more than a 3-fold and 1.5-fold increase in the PC and PA fractions, respectively, with a concurrent decrease in the CL fraction (by 40%) and St (up to 19%) (Figure 4A).

### 2.5. Fatty Acids of the Main Phospholipids under Various Conditions

**Figure 5:**
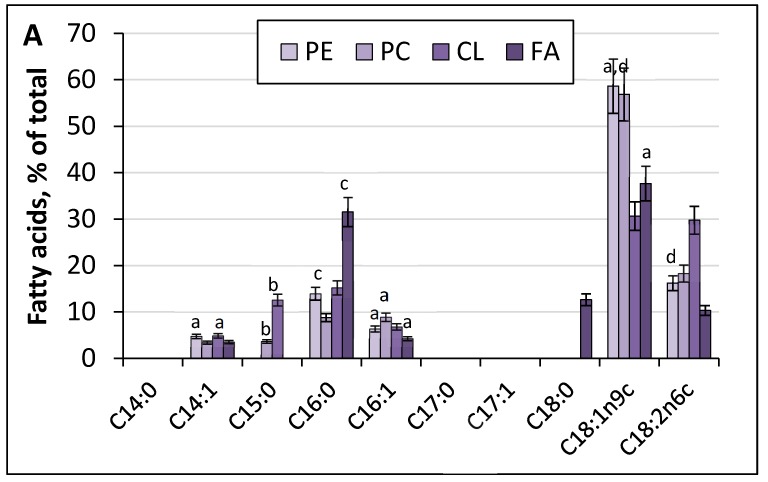

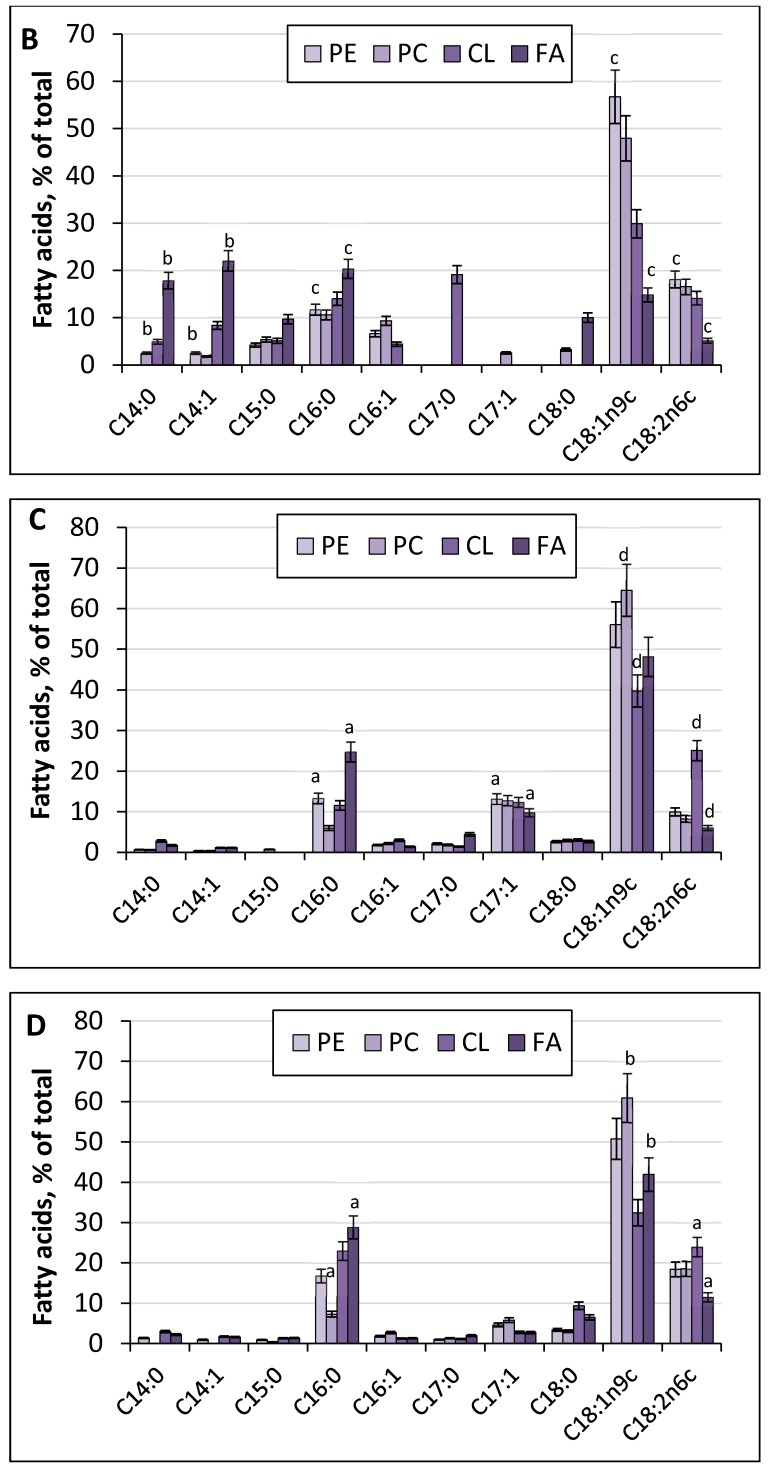

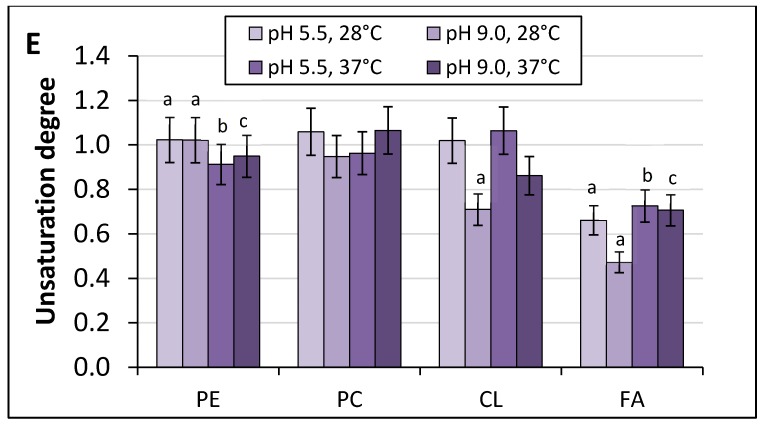
Fatty acid composition of the main membrane phospholipids of *Y. lipolytica* W29 (% of total amount). (**A**)—pH 5.5, 29 °C, (**B**)—pH 9.0, 29 °C; (**C**)—pH 5.5, 38 °C; (**D**)—pH 9.0, 38 °C; (**E**)—the unsaturation degree. a—*p* < 0.05; b—*p* < 0.001; с—*p* < 0.0004; d—*p* < 0.04.

The degree of unsaturation of acyl residues in phospholipids determines the fluidity of the membrane lipid bilayer, which in turn may influence the yeast survival and adaptation to alkaline and thermal stresses. Thus, we chromatographically isolated four major PL, the share of which comprised more than 6%, and analyzed their fatty acid composition (Figure 5). Dominating fatty acids in the main membrane PL were stearic (C18:0), oleic (C18:1), and linoleic (C18:2) ones. The PE fraction with an index of hydrogen deficiency (IHD) of 1.5 contained a lot of oleic (C18:1 n9 c) and linoleic (C18:2) acids. Under various conditions, the overall degree of acyl residue unsaturation (IHD) changed due to the alterations in the unsaturation of some membrane PL. Of note, the pH increase to 9.0 led to some (1.3 times) decreases in the fatty acid IHD of cardiolipin (CL) and phosphatidic acid (PA) fractions (Figure 5B). In the PE fraction, there was a rather high level of saturated fatty acids, namely palmitic (C16:0) (6–12%) acids. Noteworthy, growth of the culture at 38 °C and optimal pH resulted in a dramatic disappearance of myristic (C14:0) acid, which appeared again in the PC, CL, and PC fractions at alkaline pH and the elevated temperature (Figure 5C,D). The most significant changes occurred in the fatty acid composition of the CL fraction. At pH 9.0, the margaric acid content reached 14% compared to that under optimal conditions, where it was not detected.

Growth of the yeast at 38 °C was also accompanied by a decrease in the IHD of fatty acids in the membrane lipids (Figure 5E), which was mainly due to a decrease in the linoleic acid content and an increase in the oleic acid one. It should also be noted that at the elevated temperature, all the major membrane phospholipids contained margaric (C17:0) and heptadecenoic (C17:1) acids, which are usually absent in the lipid profile under optimal conditions (pH 5.5; 29 °C). Moreover, thermal shock provoked an increase in the stearic acid (C18:0) in the main membrane phospholipids, and a significant decrease or disappearance in the short-chain (C13–C15) fatty acids level in the membrane lipids (Figure 5B,D).

The combined stress caused a complete readjustment in the fatty acid composition of the dominant membrane phospholipids (Figure 5E). Besides the abundant margaric (C17:0) and heptadecenoic (C17:1) acids in the cultures grown at the elevated temperature at both pH tested, the main changes in the fatty acid repertoire concerned the ratio of C18-unsaturated fatty acids (Figure 5D,E). Unlike the *Y. lipolytica* yeast grown at optimal pH and elevated temperature, in the cells under combined stress, there was a significant increase in the linoleic (C18:2) acid content in the PE and PC fractions.

## 3. Discussion

Natural *Y. lipolytica* W29 yeast dwells in oily habitats, namely some food (cheese, yogurt, and sauces), wastewater contaminated with oils, as well as marine and hyper-mineralized sources [35,36]. Alkali- and halo-tolerance properties [1] make this organism a very promising model for biotechnological use [37]. In the present study, we could confirm the *Y. lipolytica* yeast’s successful resistance to alkaline stress (pH ≥ 8.0). *Y. lipolytica* possesses a broad optimum of the growth rate at various pH within 4.5 to 6.5 (Figure 1A). It let us render *Y. lipolytica* as a moderate alkali-tolerant [37]. This was unexpected, as it is postulated that most species of the *Yarrowia* genus grow at the temperature not higher than 32 °C [38,39]. It indicates a high thermotolerance of *Y. lipolytica* W29 grown at both pH 5.5 and 9.0.

### 3.1. Cytosol Carbohydrate Profile

Analysis of sugar compositions in *Y. lypolytica* at the optimal temperature showed that mannitol is the major soluble carbohydrate at any ambient pH tested. Polyol mannitol is reported to dominate not only in *Y. lipolytica* but also in *Yarrowia divulgata* (28 g × L^−1^, YP/S = 0.25 g × L^−1^), *Candida hollandica* (34.2 g × L^−1^, YP/S = 0.31 g × L^−1^), and *Candida oslonensis* (34.3 g × L^−1^, YP/S = 0.29 g × L^−1^) [2]. Moreover, mannitol comprises more than half of the total sugar amount in the cytosol of some alkali-tolerant fungi, namely *Acrostalagmus luteoalbus*, *Chordomyces antarcticus*, and alkaliphile species of *Sodiomyces magadii* and *S. alkalinus.* Its relative composition increased while pH lowed to acidic pH [20]. The mannitol level in *Y. lipolytica* also increased at elevated concentrations of sodium chloride [12]. Recently, mannitol has been known to be involved in quenching ROS, both in vitro and in vivo [9,14]. The protective effect of mannitol and the underlying mechanism are widely discussed. According to one of the main hypotheses, mannitol functions as a so-called “compatible” solute, which accumulates in high cellular concentrations when the organism is exposed to stress. According to another hypothesis, mannitol acts as an antioxidant [9]. Compatible compounds, including carbohydrates, polyols, amino acids, and their derivatives, have an important osmoprotective function in osmotolerant organisms, such as yeast and algae [9]. Some researchers interpret the effect of mannitol as a powerful “quencher” of hydroxyl radicals (HO^•^) due to a high reaction rate in in vitro experiments [9]. Probably, both hypotheses are correct, and mannitol can be localized in a cell at the specific site of high HO^•^ production or in the close vicinity of the key HO^•^ target molecules [9]. Based on our previous studies [40], we could suppose that constitutively high concentrations of mannitol in the *Y. lipolytica* yeast facilitated its resistance to extreme conditions by effective scavenging of ROS generated at extreme pH. No significant difference in the mannitol concentration in the culture grown at different pH (Figure 2) suggests that the role of mannitol as a factor of pH adaptation should be excluded. At both normal and alkaline ambient pH, it led to significant changes in the cytosol sugar profile. An increase in the total content of soluble carbohydrates is accompanied by the substitution of the dominant cytosol sugar for disaccharide trehalose, the significant rise in the arabitol fraction, and the disappearance of both mannitol and glucose. Under the thermal influence, the *Y. lipolytica* yeast showed that trehalose substituted the dominant mannitol in the sugar repertoire with a concurrent decrease in the glucose level. It suggests that trehalose could be either formed de novo or from mannitol to stabilize the membrane structures [8]. Trehalose is known to serve as a protector against elevated temperatures by stabilizing membrane proteins and lipids [41]. Recent studies using *S. cerevisiae* have revealed the role of the first enzyme of trehalose synthesis, trehalose-6 phosphatase synthase, in the regulation of respiration and fermentation processes. *S. cerevisiae* mutants in *tps1* exhibited impaired ethanol production, showed diminished plasma membrane H^+^-ATPase activation, and changed cell pH–homeostasis [42]. The genes encoding trehalose synthesis are known to also be present in the *Y. lipolytica* genome, but the trehalose content in the cells using either glycerol or glucose is usually below 1 nmol/mg dry weight, which could be explained by a low activity of the biosynthesis pathway and high trehalase activity [43]. However, it increased upon disruption of a gene encoding a putative trehalase or after thermal shock [43]. Thermal shock causes an increase in the trehalose level in several yeast species [44]. In *Y. lipolytica*, a thermal shock of 40 °C for 2 h increased trehalose, varying from 7 to 20 nmol/mg dry weight in different strains [37]. The data are in good agreement with our results of the trehalose accumulation as the dominant sugar in the *Y. lipolytica* cytosol at the elevated temperature. Thus, when the growth temperature increased to 38 °C, a crucial change in the cytosolic sugar profile occurred.

Nevertheless, in the present study, we showed that trehalose, as a unique component of cell adaptation to any stress, found no confirmation. Although an alkaline condition is unfavorable for *Y. lipolytica*, which is proved by increasing the superoxide anion generation as antioxidant enzyme activities under these conditions [45], no trehalose was revealed in the cells grown at the optimal temperature at both pH tested. We also observed an increase in the arabitol level at the alkaline, thermal stresses, and their combination (Figure 2B). The accumulation of *D*-arabitol and trehalose was shown for the *Debaryomyces* and *Geotrichum* fungi [46], the *Kluyveromyces lactis* yeast [47], and the *Candida albicans* yeast [10] to the exposure of thermal, oxidative, and osmotic stress factors. In the eukaryotic cells, *D*-arabitol is known as a by-product of the pentose phosphate pathway in some processes, such as a supply of the pool of NADPH-reducing equivalents [40]. It is similar to mannitol, which can be oxidized to fructose, and is also capable of reducing NADP to NADPH [16]. NADPH, in turn, participates in restoring GSSG, a universal molecule maintaining the cellular redox potential. Interestingly, the redox potential of HSSG/GSH increased up to 0.48 at alkaline pH and 0.67 at the elevated temperature compared to 0.24 under the optimal conditions (Table 1). However, the ratio of the reduced glutathione to the oxidized one decreases more than two-fold. It could indicate the detoxification of ROS under stress. The obtained data indicate the development of the anti-stress response in the *Y. lipolytica* yeast under stress conditions that could confirm this statement (Table 1). Yeast cells under different kinds of stress may generate abundant GSH, which is involved in the cell protection against the damage from O^2−^. The high level of GSH could be a marker of an acute oxidative stress [48]. The data suggest that under optimal conditions, mannitol, the synthesis pathway of which is branched from the glycolysis at the glucose-6-phosphate stage, is of great importance for the development of *Y. lipolytica* [16]. Under thermal shock conditions, the cells should, first of all, provide the membrane structure stability for the transporters to work, which is often achieved by trehalose accumulation in the cells. Since cellular trehalose is not consumed, but largely serves as a stabilizer [8], we suppose that the pentose–phosphate pathway is induced to restore reducing equivalents, which in turn leads to *D*-arabitol accumulation [46].

Our results showed that upon growth at elevated temperatures, both trehalose, which serves as an antioxidant, osmolyte, and membrane stabilizer, and arabitol, which is an osmolyte [46], play a vital role in the adaptation of the *Y. lipolytica* yeast to thermal shock.

### 3.2. Lipidome of Y. lipolytica

The results of our studies showed that glycerol-utilizing *Y. lipolytica* cells had a high lipid content. This is in accordance with the data by [49,50], which showed a high yield of synthesized intracellular lipids upon assimilation of glycerol by *Rodotorula* and *Sporobolomyces* yeast. It may be related to the fact that glycerol used as a growth carbon substrate promotes the lipid synthesis in the yeast cell. Thus, glycerol can diffuse through the cytosolic membranes of a yeast cell and then may transform into some lipids, including unsaturated fatty acids [51].

The alkaline pH adaptation of *Y. lipolytica* caused a general decrease in the storage lipid profile (Figure 3). The similar decrease in the total storage lipids at alkaline ambient pH was also demonstrated for the *Sodiomyces tronii* ascomycete [20]. The decrease in DAG levels in storage lipids under thermal and combined stresses suggested that under these conditions, the cells could use the storage lipids as substrates (Figure 3A). The data are confirmed by the results of ultra-structural changes in the *Y. lipolytica* yeast under stress with a concurrent reduction in the number and volume of the LBs (Figure 3D–F). Abundant LBs could be observed in the yeast cytoplasm (Figure 3C–F). The lipid droplets mainly participate in the storage and release of accumulated nutrients, being an energy source in the yeast cells [28,29]. Moreover, they are used as substrates to synthesize lipid components for cellular membranes. The LBs, along with some various organelles, play the key role in lipid metabolism and the maintenance of cellular energy homeostasis as well as in protein content circulation. Since the compartment stores energy, it can support and regulate the membrane composition. Besides that, it promotes lipid accumulation, resulting in an efficient adaptation and assimilation of hydrophobic substrates, like *n*-alkanes, fatty acids, and triglycerides [28,29]. The recent paper by [52] reported a stress-induced metabolic shift from fermentation to respiration, including the induction of peroxisomal *β*-oxidation of fatty acids. It stimulates mitochondrial respiration via alternative carbon sources and the activation of retrograde signaling to alleviate mitochondria damage [52]. Probably, in the case of adaptation of the *Y. lipolytica* yeast to extreme pH and elevated temperatures, a similar mechanism may work.

The amount of membrane lipids also decreased at alkaline pH (Figure 4B). The membrane lipids’ repertoire under the conditions varied slightly. However, the conditions of simultaneous pH 9.0 and an elevated temperature caused a two-fold decrease in the CL fraction of the mitochondrial membranes compared to the optimal conditions. CL is known to function as a proton trap as it has four acyl residues and two orthophosphoric acid residues, each capable of binding one proton [53]. It lets CL create a proton gradient on both the inner and outer mitochondrial membranes. CL plays a vital role in the mitochondrial functions and biogenesis of organelles by interacting with a wide range of mitochondrial proteins both via hydrophobic and electrostatic interactions and by stabilizing the mitochondrial respiratory chain proteins [54]. In 2012, Rostovtseva and Bezrukov [55] showed that CL-rich areas of the outer mitochondrial membrane manifested an increased activity of the mitochondrial porin of VDAC, which participated in scavenging the superoxide anion radical from the mitochondria. The CL fatty acids’ composition significantly influences the VDAC functions [54]. In our study, alkaline pH caused some changes in the fatty acid composition of the CL fraction. Thus, at pH 9.0, the margaric acid (C17:0) share reached 14% when compared to that at normal pH, which was equal to zero. The lauric, myristic, pentadecanoic, and palmitic acid fractions significantly decreased (Figure 5B). Earlier, Marek Kieliszek showed an increase in the margaric and heptadecenoic (C17:1) acid levels in *Candida utilis* grown either using 5% glycerol as a carbon source or enriched with selenium (20 mg/L) by two times and by 25%, respectively [28]. Also, abundant margaric (C17: 0; 12.19%) and heptadecenoic (C17:1; 9.31%) fatty acids were found when *Y. lipolytica* was cultivated in fermenters using glycerol as a carbon source. Probably, an increase in the saturated fatty acid content of the mitochondria CL fraction upon stress provides the integrity and rigidity of the cell membranes according to the homeoviscous adaptation, which includes a change in the lipid profile of the cell membrane, ensuring its necessary fluidity [56].

The elevated growth temperatures caused a significant increase in the PC fraction and a decrease in the St one in the membrane lipid profile. PC are PL synthesized from the PE in the endoplasmic reticulum via the Kennedy pathway [57]. The homeostatic equilibrium between the two classes of PL is of great importance for both performing mitochondrial functions and protecting against oxidative stress that was reported for *C. albicans*. The PC level increase was also reported for *S. cerevisiae* under stress induced by the p-HPCD detergent treatment [58]. PCs are bilayer lipids, which ensure the stability of the membrane structure [59]. Probably, in our case, both an increase in the PC fraction and a decrease in the St one could compensate for the drop in the lipid acyl chain’s unsaturation in the membranes. The fact that the fatty acids of the PC fraction at optimal temperature maintain the highest degree of unsaturation could partly confirm this speculation (Figure 5E). As far as minor membrane lipids, there was also some increase in the PA fraction in the membrane lipids under both thermal and combined stresses. The data are agree well with the results obtained by [60], where the authors using the *Aspergilus niger* fungus showed that various stressful factors (heat and cold shock, oxidative and osmotic stress) led to a significant increase in the PA level in the membranes. They speculated that the PAs contributed to the fungus adaptive defense responses to stress by increasing the cell membrane stability and by intensifying vesicular transport, and endo- and exocytosis. Probably, in the case of adaptation to thermal shock and alkaline stress in *Y. lipolytica*, a similar mechanism may occur. The changes in the IndexIHD of the acyl chains of membrane lipids depend on the kind of stress factor used. The extreme pH values affected the degree of unsaturation insignificantly, except for CL, where the total IHD of acyl chains decreased by nearly two-fold at pH 9.0 (Figure 5B,D).

In the paper by [61], the authors showed the influence of a variety of growth conditions used on the yeast lipidome, when the growth temperature led to an increase in fatty acid unsaturation as well as shortening of the chains. The overall decrease in IHD of the acyl chains of the membrane lipids under the elevated temperature could be explained by the fact that the higher unsaturation degree results in a lower melting point of the membrane. It means that it becomes much more difficult for a cell to maintain a structural organization and optimal function of cellular membranes. Similar experimental results were obtained using *Schizosaccharomyces pombe* exposed to a short temperature stress. The data demonstrated an increase in the saturated fatty acids share in the membrane lipids [56].

Interestingly, the elevated temperature and alkaline pH strongly altered both the qualitative and quantitative lipid profiles of the *Y. lipolytica* cells (Figure 3, Figure 4 and Figure 5). The data suggest that the thermal shock could manifest the most pronounced response in *Y. lipolytica*, leading to metabolic readjustments, including increased membrane rigidity, in particular, due to the appearance of some saturated fatty acids, namely penta-decanoic (C14:0) and margaric (C17:0) acids, in the lipid profile and an increase in the palmitic (C16:0) acid content (Figure 5C,D).

## 4. Conclusions

The adaptation strategy of the *Y. lipolytica* yeast to various unfavorable conditions has been studied before in [53], where the authors suggested that there was a certain general defense response to different stress factors (temperature shock, ethanol, and oxidative stress) of the yeast strain, yielding the organism the advantage of poly-extremophilicity. The adaptation mechanism includes the reduction of cyclic adenosine monophosphate (cAMP), an increase in the antioxidant enzyme activity, and induction of the alternative pathway for electron transfer in the mitochondria [62]. The adaptive strategy is non-specific and uses a general “activating center”.

Based on the experimental evidence, we conclude that the *Y. lipolytica* yeast can use different kinds of defense responses for long-term adaptation to unfavorable environmental conditions, such as alkaline pH and elevated temperature. The lipid and carbohydrate cellular profiles reflect the changes forming different and highly specific adaptive responses, accompanied by modifications of both the cell antioxidant state and oxidized and reduced glutathione levels (Table 1). Interestingly, under the cross-adaptation of *Y. lipolytica* to the combined stress, the thermal shock is evidently dominating. The elevated temperature affects the adaptive strategy of the cell, probably due to the natural alkali tolerance of the species. The changes in pH cause only some fluctuations in IHD of the lipid acyl chains while the thermal shock leads to dramatic metabolic readjustments in the sugar and lipid profiles, and in particular, the substitution of the main carbohydrates, crucial changes in the membrane lipids, and their IHD.

It is worth noting that managing the sugar and lipid composition in *Y. lipolytica* W29 can play a significant role in the application of this organism into industry. Recently, quite promising results were obtained concerning the use of the extremophile *Y.lipolytica* as a mannitol producer [62]. The authors showed that *Y.lipolytica*, grown in glycerol-containing medium at alkaline pH, enables the production of a significant amount of mannitol (27.6 g/L). It could provide the basis for developing alternative and innovative technologies of mannitol production. Mannitol is a polyol, which is in great demand by the modern food, pharmaceutical, and medical industry. The biosynthesis of polyunsaturated fatty acids using *Y. lipolytica* W29, induced in elevated temperature cultivation, can be used to produce the enriched products, which are highly sought for medical purposes.

## 5. Materials and Methods

### 5.1. Yeast Strains and Growth Conditions

Wild-type *Yarrowia lipolytica* W 29 from CIRM Levures collection (France) was used. The culture was raised in batches of 100 mL in glycerol (1%)-containing medium of the following composition (g/L): MgSO_4_—0.5, (NH_4_)_2_SO_4_—0.3, KH_2_PO_4_—2.0, K_2_HPO_4_—0.5, NaCl—0.1, CaCl_2_—0.05). Then, 2 M KP_i_ stock buffer was prepared by dissolving KH_2_PO_4_ anhydrous (272 g/L, Amresco Cat # 0781), pH adjusted with 2 M K_2_HPO_4_ to 6.0. Further, 2 M KP_i_ stock buffer was prepared by dissolving K_2_HPO_4_ anhydrous (342 g/L, Amresco Cat # 0705), pH adjusted with 2 M KH_2_PO_4_ to 9.0. Both KP_i_ buffers were sterilized by autoclaving and added to sterilize the culture medium (ratio 1:40) just before inoculation. The yeast was cultivated at different ambient pH from 3.0 to 9.0 on a rotary shaker at 150 r.p.m at temperatures of 29 and 38 °C as described in [63]. Absorbance (A) was assessed in cell suspension at the wavelength of 590 nm (A_590_) using a Specol-11 spectrophotometer (Germany). The yeast was raised in the stationary growth phase.

### 5.2. Cell Viability and Vitality Assays

To determine culture viability, the yeast cells from the stationary phase were centrifuged, washed with sterile water, and suspended to the final density of 10^8^ cells mL^−1^ in 100 mM KP_i_ buffer; pH 7.0. To determine cell viability and vitality, the following methods were used [64].

#### 5.2.1. Spotting Test

Cells were suspended in sterile water and diluted to give 10^5^, 10^4^, or 10^3^ cells mL^−1^. Samples (10 µL) of each suspension were inoculated on solid YPD medium with pH 5.5 and 9.0 and incubated at 29 °C. Colony growth was inspected after 48 h.

#### 5.2.2. Staining with Methyl Blue

Yeast cells were suspended in phosphate-buffered saline (PBS), and a 200-µL sample of the cell suspension was mixed with 100 µL methylene blue (0.1 mg mL^−1^ stock solution, dissolved in a 2% dihydrate sodium citrate solution) and incubated for 5 min at room temperature. Viability was examined under a light microscope using Gorjaev’s chamber (×400) from at least 1000 cells in one biological replicate. Viable cells were colorless, and dead ones were blue.

### 5.3. Potential-Dependent Staining

Potential-dependent staining of mitochondria in the *Y. lipolytica* cells using Rh123. Cells were incubated with 0.5 µM Rh123 and examined in 30 min. The incubation medium contained 0.01 M PBS, pH 7.4, and 1% glycerol. Regions of high mitochondrial polarization are indicated by red fluorescence due to the concentrated dye. To examine the Rh123-stained preparations, filters 02, 15 (Zeiss) were used (magnification ×100). The photos were taken using an AxioCam MRC camera [65].

### 5.4. Transmission Electron Microscopy (TEM)

TEM analyses of the *Y. lipolityca* cells were performed as described previously [66] and were examined using Jeol (JEM-100 B; Tokyo, Japan) and Hitachi U-12 (Tokyo, Japan) electron microscopes.

### 5.5. Preparation of Cellular Homogenate

The cellular homogenate was obtained as follows: Cells were washed twice with ice-cold water, and resuspended in grinding medium (1:1 *w*/*v*). The medium contained: 10 mM MES, 0.5 M mannitol, 5 mM EDTA, and 0.5 mM phenyl-methylsulfonyl-fluoride (PMSF); pH 6.5. The yeast cells were disrupted with an ultrasonic disintegrator 9 MSE (Farmacia, Sweden) using some pulses at 0 °C for 2 min interrupted by cooling periods every 30 s. The obtained homogenate was centrifuged at 10,000× *g* for 30 min and the supernatant was collected for further experiments [65].

### 5.6. Enzymes Activities Assay

Total catalases (CATs) and superoxide dismutases (SODs) activities in cell suspension were assessed according to [65].

### 5.7. Glutathione HPLC-ECD Analysis

The harvested yeast cells were briefly washed with deionized water and frozen in liquid nitrogen for further HPLC analysis. Then, 100 µL of re-frozen yeast lysate was added to 500 µL of 0.1 M cold iced perchloric acid (PCA). After brief vortexing, suspension was sonicated for 5 s and placed on ice for 10 to 15 min for better metabolite extraction. The obtained cell homogenate was centrifuged twice for 20 min at 14,000 rpm in precooled (+4 °C) micro centrifuge. Then, 200 µL of clear supernatant was loaded into HPLC vial for direct HPLC analysis. The HPLC system for glutathione measurements was equipped with a CouloChem-III electrochemical detector, Waters 717 plus auto-sampler with a cooled platform (+4 °C) and Waters 515 HPLC pump. In total, 20 µL of mobile phase (0.1 M LiH_2_PO_4_, 1.5 mM octenylsuccinic anhydride, and 7% methanol) was delivered at flow rate of 1.0 mL/min in an isocratic mode. Pre-column was Super ODS A0114; 4.6 mm × 5 cm, particle size 2 µm and analytical column was ESA HR-80; 80 mm × 4.6 cm, P/N 68-0100 with particle size 3 µm and pore size 120 A. Both columns were maintained at +30 °C. Under the conditions, GSH eluted at 1.61 min and GSSG at 2.72 min. Sample processing, files storage, and data analysis were controlled by EZChrom Elite software (Dionex & Thermo Fisher Scientific Company, Bedford, MA, USA). The concentration of GSH and GSSG was calculated based on a calibration curve equation per mg of protein.

### 5.8. Preparation and Analysis of Lipids

To determine cell lipids, yeast cells in the stationary growth phase were raised and centrifuged at 6000× *g*, washed twice with cold distilled water, and frozen liquid nitrogen [67]. The weighted sample of about 1 g was immediately homogenized in isopropanol to de-activate lipases by pestle and mortar, and incubated at 70 °C for 30 min. Then, the biomass was homogenized once more using some sand and the lipids were extracted by the method described in [67], which involved extraction with isopropanol and the isopropanol–chloroform mixture (1:1 and 1:2) at 70 °C, evaporation in a rotary evaporator, and extraction of the residue with chloroform-methanol (1:1) supplemented with 5% sodium chloride solution and water to remove water-soluble substances. After separating the mixture with a vortex, we dried the chloroform layer by passing it through water-free sodium sulphate, evaporated, and desiccated with a vacuum pump. The resulting pellet dissolved in a small amount of chloroform-methanol (1:1) was stored at −21 °C. The composition of storage lipids was assayed using an ascending thin layer chromatography on glass plates with silica gel 60 (“Merck”, Darmstadt, Germany). To separate storage lipids, the hexane:sulphuric ether:acetic acid (85:15:1) system [68] was used. To separate phospholipids and sphingolipids, SI60 Silica thin layer chromatography plates were activated and developed in two dimensions, first with chloroform/methanol/water (65/25/4, by volume) and second with chloroform/acetone/methanol/acetic acid/water 950/20/10/10/5, by volume) [69]. The lipids (100–200 μg) were applied to a plate bovine serum glycoceramides, and PC used as sphingolipid (SL) and phospholipid (PL) standards were applied on chromatograms before passing in the second direction. Samples of SL (5 and 10 µg) and PC (10 and 20 µg) were applied on the plates. To develop the stains, the chromatograms were sprayed with 5% sulfuric acid in ethanol, followed by heating up to 180 °C. To detect phospholipids, the developed thin-layer plates were sprayed with 0.1% (*w*/*v*) ninhydrin for the ones carrying free amino groups and with *α*-naphtol for glycolipids. Lipids containing a quaternary ammonium group were visualized at room temperature with Dragendorff’s spray reagent [70]. Sphingolipids were detected in the glycolipid fraction by the saponification method [71]. Storage lipids were identified with individual markers for di- and triglycerides (DAG, TAG), sterols (ergosterol), free fatty acids (FFA), and hydrocarbons (“Sigma”, Sant Louis, MO, USA). Quantitative analysis of the lipids was performed using the Dens software (“Lenchrom”, S-Peterburg, Russia). Thin-layer chromatography (TLC) plates of storage lipids were added to the Supplementary (Figure S1). To assess the fatty acid composition of PL, separate PL were isolated using chromatography with two plates, and eluted with chloroform/methanol (1/1, *v*/*v*) for a night. Then, the supernatant was decanted, evaporated, 1 mL toluene and 2 mL of 2.5% H_2_SO_4_ dissolved in methanol, and kept for two hours at +70 °C. Fatty acid methyl ethers were extracted with hexane, dried, and analyzed by a Kristall 5000.1 gas chromatograph (“Chromatek”, Yoshkar-Ola, Russia) using an Optima-240 (60 m × 0.25 mm) capillary column (“Macherey-Nagel GmbH & Co.”, Duren, Germany). The temperature program was set from +130 to +240 °C. Eluting fatty acids were identified using the Supelco 37 Component FAME Mix (a mixture of fatty acid methyl esters) (“Supelco”, Sant Louis, MO, USA).

### 5.9. Carbohydrate Analysis

The sugars were obtained by repeated four-time extraction with boiling water for 20 min. Proteins were removed from the resulting extract [70]. The extract was further purified of charged compounds using a combined column with the Dowex-1 (acetate form) and Dowex 50 W (H^+^) ion exchange resins. The sugar composition was determined by gas-liquid chromatography using trimethylsilyl sugar derivatives obtained from the lyophilized extract [71]. *α*-Methyl-*D*-mannoside (“Merck”, Darmstadt, Germany) served as the internal standard. Chromatography was performed with a Kristall 5000.1 gas chromatograph (“Chromatek”, Yoshkar-Ola, Russia) equipped with a ZB-5 30 × 0.32 mm capillary column (“Phenomenex”, Torrance, CA, USA) temperature program was set at +130, 5–6 °C/min gradient to +270 °C. Glucose, mannitol, arabitol, inositol, and trehalose (“Sigma”, Sant Louis, MO, USA) served as the standards.

### 5.10. Statistical Analyses

Data are presented as the average ± standard deviation in biological triplicates with a standard error of less than 5%. Analysis of soluble carbohydrates and lipids was performed using one-way ANOVA (*n* = 3). *p* values were determined by the two-tailed paired t-test at the 5% level of probability.

## Figures and Tables

**Table 1:** Redox state of *Y. lipolytica* yeast cells.

Antioxidant Compound	Growth Conditions			
	pH 5.5; 29 °C	pH 9.0; 29 °C	pH 5.5; 38 °C	pH 9.0; 38 °C
SODs *	141 ± 11 a	831 ± 48 a	1725.48 ± 63 a	1407.13 ± 55 a
CATs **	26.58 ± 5.67 b	21.7 ± 1.78	2.33 × 10^7^ ± 1.12 × 10^6^ b	4.59 × 10^5^ ± 2.59 × 10^4^ b
[GSH] ^†^	15.22 ± 1.31 c,d	8.68 ± 0.75 c	54.90 ± 4.22 d	23.30 ± 3.15
[GSSG] ^‡^	3.62 ± 0.25 e,f	4.15 ± 0.56	36.70 ± 4.01 e	11.39 ± 1.32 f
[GSSG]/[GSH]	~0.24	~0.48	~0.67	~0.48

* Values are mean ± SEM (unit of enzymatic activity of SOD per 1 mg of protein) from 5 to 6 independent experiments; ** Values are mean ± SEM (total CATs activity are μmols H_2_O_2_ per 1 mg of protein) from 5 to 6 independent experiments. ^†^ Values are mean ± SEM (total GSH content in μM/mg *w*/*w*) from 5 to 6 independent experiments. ^‡^ Values are mean ± SEM (total GSSG content in μM/mg *w*/*w*) from 5 to 6 independent experiments. a—*p* < 0.01; b—*p* < 0.0004; c—*p* < 0.03; d—*p* < 0.003; e—*p* < 0.004; f—*p* < 0.001.

## Supplementary Materials

The following are available online at https://www.mdpi.com/2218-1989/9/12/307/s1, Figure S1: TLC plates of storage lipids.
