# Supplementary figures and images for: Soluble Sugar and Lipid Readjustments in the Yarrowia lipolytica Yeast at Various Temperatures and pH
Source: Metabolites. 2019 Dec 17;9(12):307. doi: 10.3390/metabo9120307 (PMC6950712; doi:10.3390/metabo9120307)

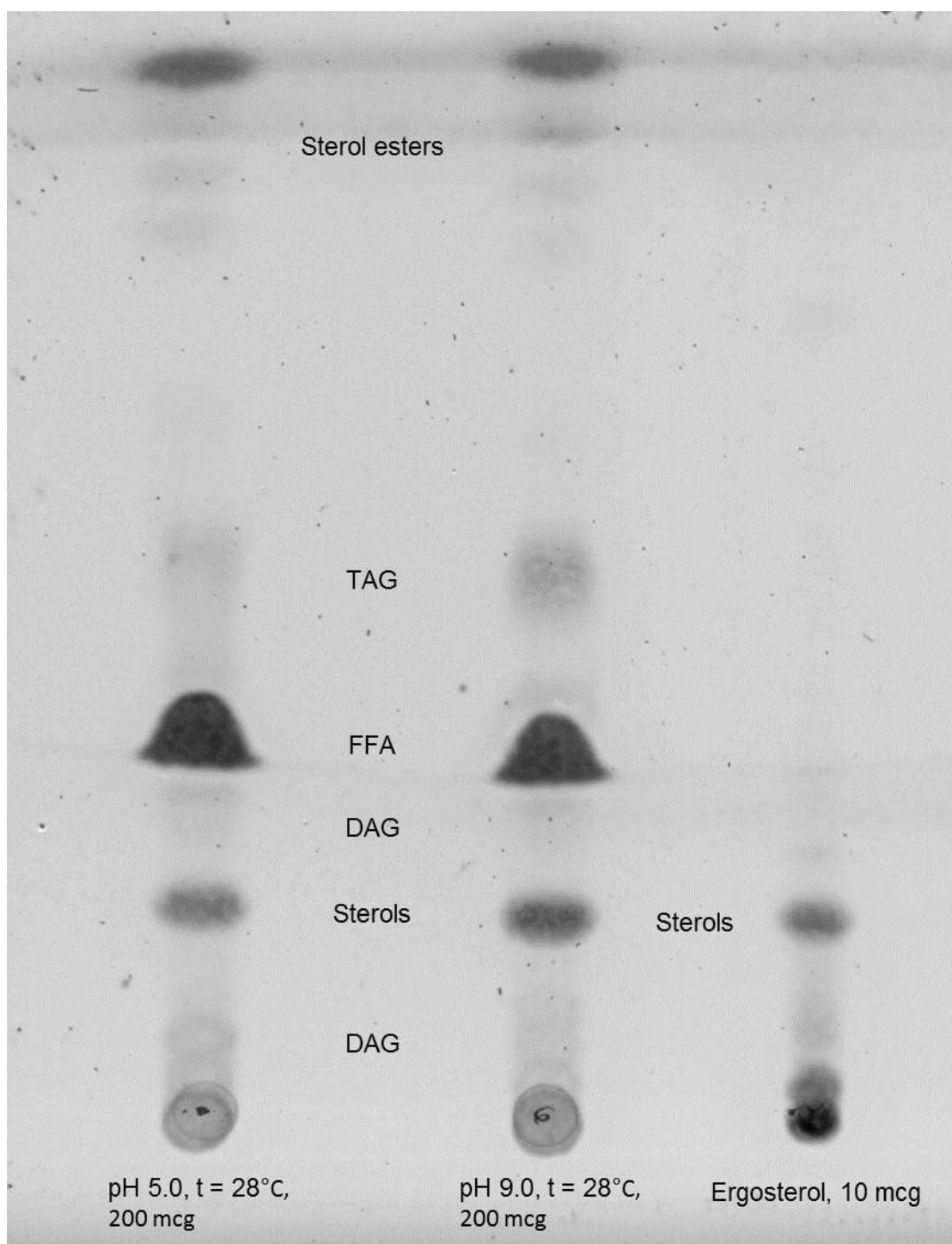

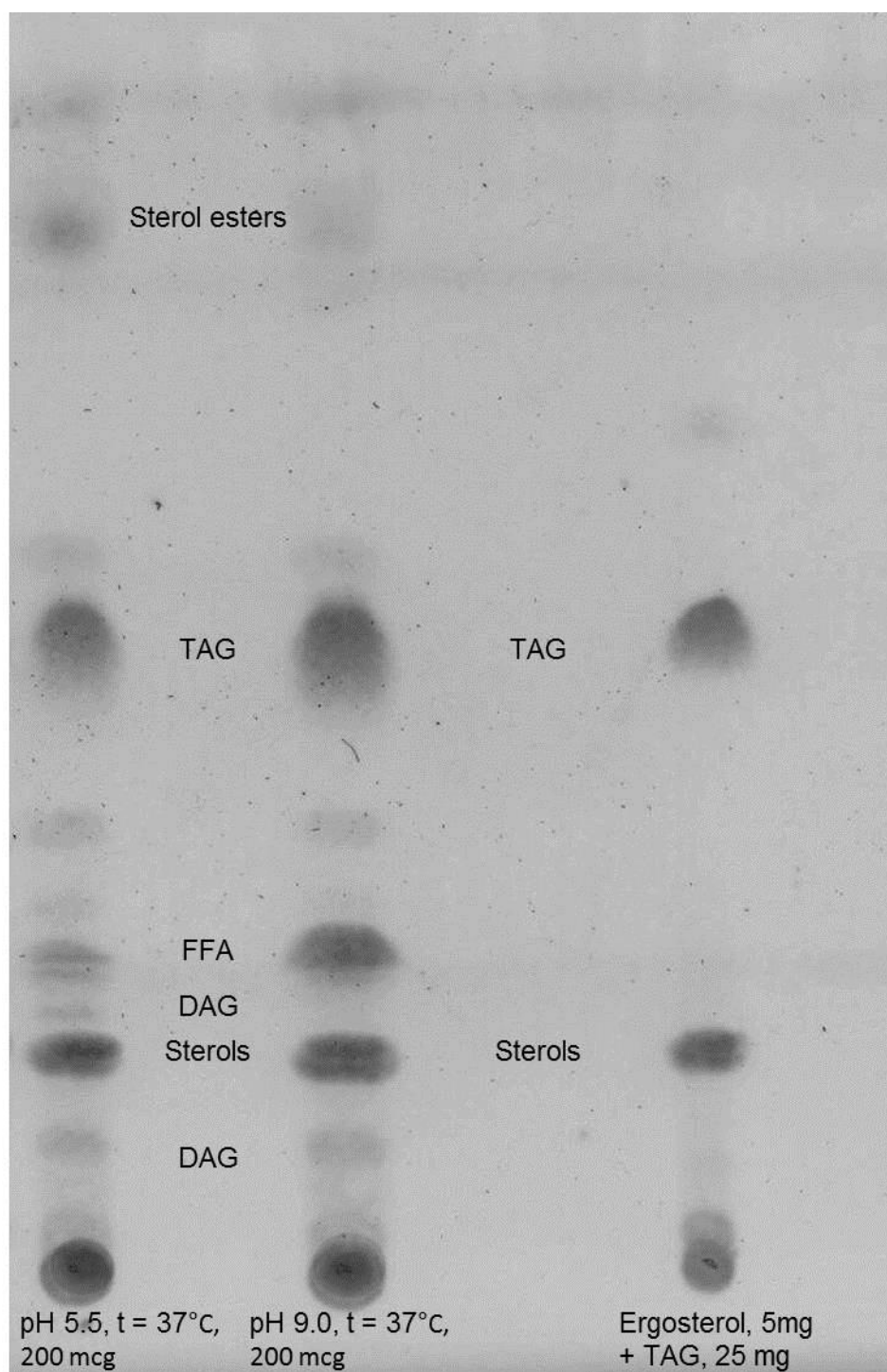

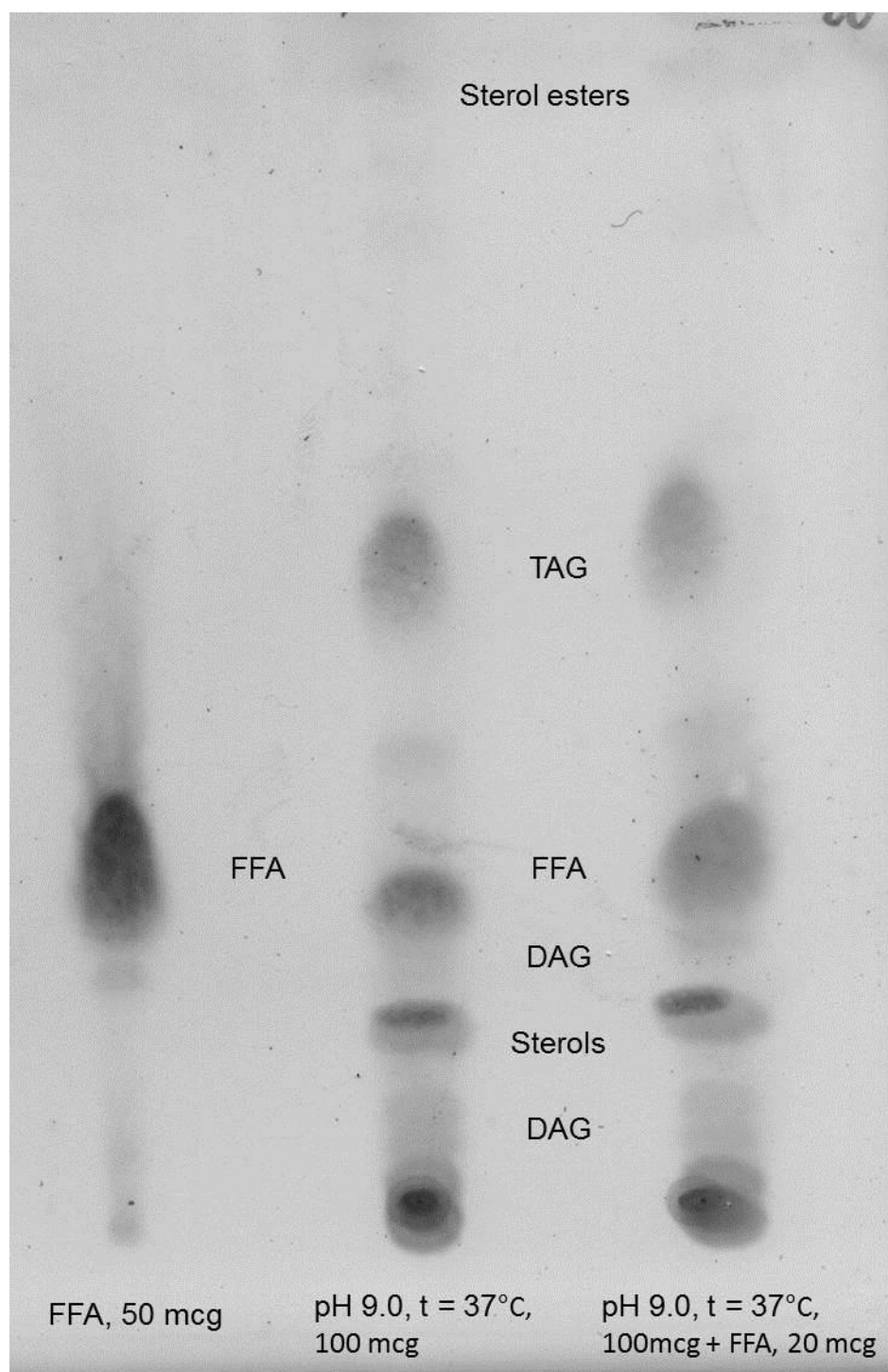

Supplement: Supplementary file 1 [file metabolites-09-00307-s001.pdf]
